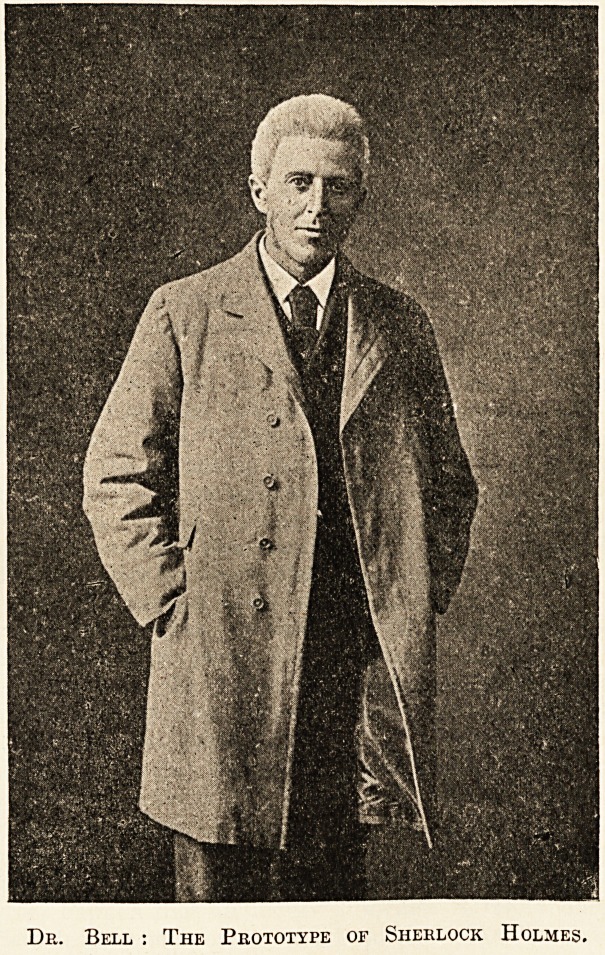# The Passing of a Great Teacher

**Published:** 1911-10-14

**Authors:** 


### THE MAKING OF THE MODERN PRACTITIONER.

### The Passing of a Great Teacher.

The modern medical practitioner, consultant, specialist, general practitioner, or what not, is essentially the product of his training; that is, of the courses of study as controlled and inspired by the General Medical Council, and of the methods of carrying them out in the particular school where he has been taught. Environment has much, indeed, to do with his professional capacity and efficiency; and that environment, since the abandonment of the old apprenticeship system, has necessarily tended to become in some ways stereotyped. This is inevitable in large schools of young men all studying the one subject—as opposed to the more diverse intellectual life of a university, where many courses of study are conducted.

Does it follow, then, that the medical practitioner runs a risk of becoming too much the creature of his environment, too much a mechanical craftsman and too little an original thinker? Though the danger cannot be denied, it is in reality not a pressing one, or at any rate not a danger that any young man in the medical profession need fall into if he wishes to avoid it. One reason for this is the immense diversity of human life, human nature, and human illness as it reveals itself to the doctor at the bedside, in the consulting room, or in the wards of a hospital. *Nihil humani a me alienum puto* is a motto which, consciously or not, every doctor has to act up to; and it is this contact with humanity, in all its strength and all its weakness, that should, and generally does, preserve a wholesome humanity and a freshness of outlook on all his fellow creatures. Character, in fact, is as important as ever it was—probably more important than ever—for success in medical practice; and he who forgets to study his patients along psychological as well as purely physical lines is little likely to develop that personal magnetism which seems to imbue all really great clinicians.

If character is so essential to the making of a successful doctor in practice, how vastly more is it required of a successful teacher! And be it remarked that, curiously, the two things do not necessarily go together; that is, a man with a gift for teaching has not necessarily in an equal degree the gift of clinical acumen or therapeutic sagacity. Still, these two valuable qualities are often combined in the same man; and a notable example of the ability of a man of real character to inspire his pupils has just been recalled to the profession by the death on October 4 at the age of seventy-four of Dr. Joseph Bell, famous even more as the prototype of Sherlock Holmes than as a successful teacher and an all-round surgeon. As a testimony to his merits in a strictly professional respect, his record speaks for itself. Consulting surgeon to the Edinburgh Royal Infirmary, after passing through every intermediate grade from that of dresser upwards; demonstrator of anatomy in the University of Edinburgh; ex-president of the Edinburgh Medico-Chirurgical Society and of the Scottish Medical Association; consulting surgeon to the Edinburgh Eye Infirmary and to the Edinburgh Royal Hospital for Sick Children; and editor for twenty-three years of our valued contemporary, the *Edinburgh Medical Journal*. Such an all-round record is an impossibility now, but even in Dr. Bell's day it can have been won only by unsparing work and energy, to say nothing of most unusual abilities.

Dr. Bell: The Prototype of Sherlock Holmes.

It was as a teacher at Edinburgh that Dr. Joseph Bell's character and attainments as an observer were displayed before the admiring eyes of Mr. (now Sir) A. Conan Doyle; and it was the former's power of rapid and certain inference, founded on precise and informed observation, that suggested to the latter the leading characteristics of Sherlock Holmes. Sir Arthur Conan Doyle, once a general practitioner himself, has himself recorded Bell's power of diagnosing much of the previous history, occupation, habits, and character of the Infirmary patients from some minute but significant trait which to others would have meant nothing and would probably have passed unobserved. Some peculiarity of gait, some slight manual abnormality, some unusual shade of complexion, would offer a clue as to occupation; the disease of the patient might give a further hint, and the patient's manner of speech and demeanour might complete a chain of evidence which enabled the surgeon to astound his hearers by amazing inferences of such kind as those presented in the Adventures of Sherlock Holmes.

After all, this method of observation is that on which every diagnosis is based. The collection and consideration of every available fact; the rejection of one explanatory hypothesis after another, until only one disease remains which can account satisfactorily for every sign and symptom; that is the method which every medical student is taught, though it is not every teacher who observes as closely as Dr. Bell, or has an encyclopædic knowledge of his profession. Nor is the method a modern development of scientific education. Zadig, as readers of Voltaire know well, pursued it long before medicine had emancipated itself from those superstitions which the great French satirist was wont to castigate. Dr. Bell would have been the last to claim any originality in the principle of his method, though certainly it would appear he carried that principle to logical conclusions which others never thought of. The interest of his career, now closed for ever, lies chiefly in the manner in which it illustrates the foundations of success in diagnosis. First, a complete knowledge of the whole of the signs and symptoms of every morbid state, as far as at present research has revealed them; secondly, a sedulous collection by trained observation of every sign or symptom exhibited by the patient; thirdly, the exclusion of every disease which the patient can clearly be proved not to have; and lastly, if more than one disease remains, a suspension of judgment until more facts can be observed to clinch the matter. That is the only scientific method of medical diagnosis; and it is exactly the same method which, applied to crime and criminals, made the reputation of Sir A. C. Doyle and of Sherlock Holmes.

Notice to Correspondents.—The points raised by Mr. Buchanan and Mr. Hamilton will be dealt with fully next week.

## Figures and Tables

**Figure f1:**